# Supportive Home Environments as a Mediator Between Sensory Impairment and Disability

**DOI:** 10.1093/geroni/igaa057.696

**Published:** 2020-12-16

**Authors:** Bernard Steinman, Jon Pynoos, Casandra Mittlieder, Julie Overton, Treva Sprout Ahrenholtz

**Affiliations:** 1 University of Wyoming, Laramie, Wyoming, United States; 2 University of Southern California, Los Angeles, California, United States

## Abstract

Home environments are important to older adults and can help to preserve independence in every day functioning when they are supportive. Nevertheless, over time, the quality of “fit” between individuals and their homes can decrease because of age-related physical changes. A significant proportion of older Americans experience sensory impairments that impact their capacity to perform daily living activities necessary to remain independent at home. Home environments designed to support access and safety have potential to ameliorate disability associated with declines in sensory status. Data from the National Health and Aging Trends Study (NHATS) were analyzed to assess the role of supportive home environments in mediating the relationship between self-reported measures of vision impairment, hearing impairment, and dual vision-hearing impairment and related ADL/IADL outcomes in community-dwelling older adults. Guided by the International Classification of Functioning, Disability and Health (ICF), regression models included covariates for sociodemographics, chronic conditions, mobility functioning, and participation. Supportive home environments were operationalized using indicators of whether participants had access to homes from the outside without having to use stairs; presence of a bedroom, kitchen, and full bathroom with a shower or tub on the same floor; and whether bathroom fixtures had been modified with features such as grab bars. Results suggest a statistical relationship between sensory function and disability that is explained in part by the lack of supportive home features. Implications are that older adults with sensory impairments can benefit greatly by improving environments in areas of the home that are known to cause difficulty.

